# Enhancing Nutrition Care in Primary Healthcare: Exploring Practices, Barriers, and Multidisciplinary Solutions in Ireland

**DOI:** 10.3390/ijerph22050771

**Published:** 2025-05-13

**Authors:** Ebipade Juliet Eyemienbai, Danielle Logue, Gemma McMonagle, Rónán Doherty, Lisa Ryan, Laura Keaver

**Affiliations:** 1 Department of Health and Nutritional Science, Atlantic Technological University, F91 YW50 Sligo, Ireland; 2 Regional Hospital Mullingar, Health Service Executive, N91 NA43 Westmeath, Ireland; 3 Department of Tourism and Sport, Atlantic Technological University, F92 FC93 Letterkenny, Ireland; 4 Department of Sport, Exercise and Nutrition, Atlantic Technological University, H91 T8NW Galway, Ireland

**Keywords:** dietary counselling, multidisciplinary team, nutrition care, barriers and facilitators, primary healthcare professionals, perceived role, training needs

## Abstract

Good nutrition promotes a healthy population and mitigates the risk of disease. Integrating nutrition care in the primary healthcare system is considered an essential plan of action to manage poor nutritional status in the population. The role of primary healthcare professionals (HCPs) in the delivery of nutrition care is especially crucial due to a current lack of dietitians and dietary support in the primary care setting in Ireland. This qualitative research explored the current practice, barriers, facilitators, and feasible solutions proposed to optimize the provision of nutrition care by primary HCPs. Twenty semi-structured interviews (pharmacists (n = 14), dietitians (n = 3), a physiotherapist (n = 1), a speech and language therapist (n = 1), and a healthcare assistant (n = 1) were conducted. Six themes were derived from the data: current practice of nutrition care in primary care, perceived role, barriers and facilitators, the importance of a multidisciplinary patient-centred approach, training needs and preferences, and addressing barriers. Participants acknowledged the importance of nutrition care in clinical practice, the principal role of the dietitian as part of the multidisciplinary team, and the essential clinical competencies and nutrition training models that may facilitate the provision of nutrition care in primary practice. A paradigm shift to a multidisciplinary care model that prioritises the integration of nutrition care into primary care practice to ensure optimal dietary counselling is afforded to patients is essential.

## 1. Introduction

Poor dietary choices and/or unhealthy eating habits contribute to global mortality [1]. According to the World Health Organisation (WHO), approximately 80% of non-communicable diseases “could be averted by adopting a nutritious diet, engaging in regular physical activity, refraining from tobacco use, and consuming alcohol in moderation” [2]. Nutrition care refers to “any range of practices with the aim to improve the dietary habits and intake of patients geared towards achieving optimal health outcomes through the inclusion of nutrition assessment, advice or nutrition counselling” [3,4]. Optimal patient-centred care necessitates a paradigm shift in disease management from a traditional clinician-based care model to one that endows patients with the ability to make shared decisions and be equipped with the right information to meet their care needs [5,6,7]. In spite of the strong clinical evidence citing enhancements in the patients’ clinical outcomes, nutrition care is sub-optimally incorporated into multidisciplinary practice in clinical practice and primary care [8,9].

Primary care encompasses a wide consortium of services delivered by general practitioners (GPs), dentists, public health nurses (PHNs), community welfare officers, community pharmacists, general and practice nurses, chiropodists, registered dietitians, midwives, social workers, community mental health nurses, physiotherapists, occupational therapists (OTs), healthcare assistants (HCAs), speech and language therapists (SLTs), psychologists etc. [10]. Dietitians are the primary providers of nutrition care as part of this multidisciplinary team (MDT). However, evidence shows that improved competency in the provision of nutrition care by other healthcare professionals (HCPs) will improve patient outcomes and help manage the global decline in dietetic support [11,12]. At the primary care level, there are long waiting lists and limited access to nutrition care and counselling, while some remain unaware that this service exists [13,14,15]. In Ireland, individuals visit their GP about 4.34 times per year, with the average consultation lasting approximately 13.7 min [16]. Nutrition-related conditions are estimated to account for over a quarter of all visits to primary care providers [17]; therefore, the integration of nutrition into these consultations could be beneficial, not as a replacement for dietitians but as a point of entry in the system.

Due to the multidisciplinary element of primary care, primary healthcare professionals are ideally situated to provide interventions on dietary counselling, behaviour change, and physical activity [18,19]. Integrating nutrition care in the primary healthcare system is considered an essential plan of action to manage poor nutritional status in the population [12,20]. Despite this, nutrition knowledge, training, and education received by primary HCPs remain suboptimal [21,22,23]. Unlike the UK, the US, Canada, Australia, and New Zealand, Ireland does not have specific guidelines for incorporating nutrition competencies into medical undergraduate programs [24]. Despite their potential role in helping patients manage chronic conditions, HCPs, with the exception of dietitians, receive minimal nutrition training [25].

In various healthcare settings, practitioners acknowledge the importance of dietary counselling but often lack confidence in providing dietary counselling and perceive it as outside their role [26]. Barriers to providing nutrition care include time constraints, inadequate training, and a lack of pathways to referral to allied health professionals [27,28]. These barriers may result in missed opportunities for dietary counselling in patient management and consequently result in poor clinical outcomes in some patients. In Ireland, HCPs cite time constraints, lack of resources, and insufficient knowledge as the primary barriers to offering dietary counselling [29]. While much research has focused on identifying these barriers, there is a lack of investigation into the perceived facilitators of providing dietary counselling. Studies have also largely reported on the provision of nutrition care and competencies in healthcare professionals in an acute setting, as well as medical practitioners, physiotherapists, and pharmacists more generally, excluding a large cohort of other primary HCPs. This study aims to examine the perceived role, barriers, facilitators, and training needs essential to the provision of nutrition care by primary HCPs in the Republic of Ireland.

## 2. Materials and Methods

Ethical Approval

This study was conducted in accordance with the Declaration of Helsinki [30], and ethical approval was granted by the Department of Health and Nutritional Sciences Research Ethics Committee, Atlantic Technological University, Sligo (2023PHN01).

Study Design

This qualitative study utilised semi-structured interviews and has been reported according to the consolidated criteria for reporting qualitative research (COREQ) [31].

Participants

Participants were primary HCPs, aged ≥18 years, who spoke English and worked and resided in the Republic of Ireland.

Data Collection

An email outlining the study was circulated to primary HCP organisations, societies, and workplaces, in addition to being shared on social media through a project-specific X (Twitter) profile. Those who were interested could click on the link provided and be brought to a Microsoft Forms page with a participant information leaflet, additional study information, and researcher contact details if there were any unaddressed questions. Finally, they could provide informed consent, complete some demographic questions around gender, age, healthcare profession, and any nutrition training, in addition to preferred interview days and times, as well as follow-up details to schedule an interview.

Interviews were conducted using Microsoft Teams, and verbal consent was reconfirmed and appropriately documented at the start of each interview. One member of the research team conducted the interview while a second researcher observed and took notes. The interview topic guide can be found in the Supplementary Materials, and questions were based on those used in a recent study on attitudes, work roles, and barriers to nutrition care in Australian and UK-based medical doctors [26]. The interview duration was 34.5 ± 13.5 min (Mean ± SD). These recordings were transcribed verbatim within one week of the interviews taking place and then deleted. After the interview, participants were informed of the one-week period following their interview to withdraw their data from the study, after which point it would be unidentifiable. Transcripts were not sent to the participants. There was no prior relationship between the researchers and the participants.

Thematic Analysis

Inductive thematic analysis using a “six-phase” analysis process: (1) data immersion, (2) data familiarisation, (3) code generation, (4) selecting themes, (5) thematic review, and (6) final themes selection, was undertaken. The research was not based on any specific theory or hypothesis; rather, the data collected were analysed to examine what themes were generated [32].

Two researchers independently coded one of the transcripts, then met for comparison and discussion with the team, and a high level of agreement was found. The two researchers continued to code and compare the remaining transcripts to ensure consistency throughout. Once complete, the team met to review these codes, discuss, and finalise themes and supporting quotations.

## 3. Results

Twenty individual semi-structured interviews were conducted over a 4-month period (see Supplementary Materials for Microsoft Forms questionnaire and semi-structured interview questions). A total of 75 potential participants completed the consent form, but 55 were absent for their scheduled interview. The video recordings were transcribed and included in the thematic analysis.

There were 15 female and 5 male participants. Practice experience ranged from 1–30+ years post license/registration, with 60% having ten or less years’ experience. Table 1 presents a summary of the participants’ characteristics.

From the thematic analysis, six main themes were identified: (1) current practice of nutrition care in primary care; (2) perceived role of the HCP in the provision of nutrition care; (3) barriers and facilitators to the provision of nutrition care in primary care; (4) importance of a multidisciplinary and patient-centred approach in the provision of nutrition care; (5) training needs and preferences to improve competency in nutrition care; (6) addressing barriers to the provision of nutrition care in primary care.

Current practice of nutrition care in primary care

All participating dietitians acknowledged routine practice of dietary counselling as their main role, and other HCPs reported varying levels of practice, which correlated with their years of experience and scope of practice. Most participants reported the provision of nutrition care/dietary counselling in some capacity to the patients/service users attending their service. Variability in the provision of dietary counselling ranged from “infrequent counselling”/“casual counselling” to “regular counselling”. The majority of the participants reported offering more generalised advice, while the remaining participants offered more “need-based”/“personalised advice”. These tended to be pharmacists with more years of experience and dietitians. The generalised advice included advice on protein, calorie, and energy requirements, healthy and unhealthy food, balanced diets, nutrient supplementation, and behaviour change models. Participants who reported proffering need-based and personalised advice mentioned chronic conditions like hypertension, elevated serum cholesterol, and diabetes.

“As a community pharmacist, I did this for years and part of my daily role was provision of nutritional advice or advice regarding vitamins deficiencies, how to correct them, how to use supplements. So, that was part of my daily job”—P7 (Female, Pharmacist, 10–20 years’ experience).

“It’s definitely something I discuss with patients and I’m not sure how comfortable I feel giving specific advice. But I would delve into it definitely. It would be more general advice. I suppose it’s probably feels somewhat outside of my scope of practice to be giving specific advice on specific and say conditions or anything from a dietary perspective”—P4, (Physiotherapist, 5–10 years’ experience).

Perceived role of the HCP in the provision of nutrition care

Three sub-themes were identified under this theme: (1) general role; (2) duty of care; and (3) the role of the dietitian. Participants reported that a combination of factors, including the national shortage of dietitians, the increasing difficulty in securing GP appointments, and the ease of accessibility to primary care facilities, has made them the first point of contact for queries regarding nutrition, nutrition supplementation, and the management of chronic conditions. The participants acknowledged the deficits in the onward referral system from the hospital to primary care and the systemic gaps in the care structure in Ireland, therefore positioning themselves as the gatekeepers and point of entry into the healthcare system.

“If there’s a dramatic weight gain or loss, it can have a massive effect on how those medications are going to work. So we would be like a gatekeeper in a way”—P1 (Male, Pharmacist, 30+ years’ experience).

“I feel obligated because there is a lack of access to dietitians for a lot of the families I work with”—P17 (Female, speech and language therapist (SLT), 5–10 years’ experience).

The second sub-theme was the participants’ duty of care to their patients and service users. Some participants acknowledged their limitations in delivering nutrition care, but owing to their duty of care to address all care needs of their patients, they would engage in more research to ensure safe and holistic practice. Pharmacists who regularly engage in the dispensing of oral nutritional supplements reported that their knowledge of nutrition care stemmed from patients’ prompts and holistic approaches to helping patients.

“I believe we have a duty of care to make sure that we understand some of the inputs including nutrition, that impact the efficacy of the medicine that is prescribed and its impact on the diseases that they are being treated for and the prevention of diseases in the future”—P9 (Female, Pharmacist, 20–30 years’ experience).

“As a pharmacist, because we in the Community Pharmacy, you can buy a lot of nutritional supplements and as a healthcare professional we are obligated to have knowledge about them and provide this information to the patients who are coming to the pharmacy and ask for this information”—P6 (Female, Pharmacist, 10–20 years’ experience).

The third sub-theme that was identified is the role of the dietitian. Most participants agreed that the responsibility of managing patient care in relation to nutrition and dietary counselling lies with a dietitian. The dietitians who participated in the study affirmed this responsibility.

“I’m a dietitian, and so, nutrition care would be the primary role, I see that as my role and my responsibility, and children are referred to me for that purpose.”—P7 (Female, Dietitian, 1–5 years’ experience).

“I suppose I’m a dietitian, so it would be the main role. Like the main kind of place they might come for the advice.”—P3 (Female, Dietitian, 1–5 years’ experience).

Barriers and facilitators to the provision of nutrition care in primary care

Participants reported limited consultation time, a lack of training and additional qualifications in nutrition, gaps in clinical knowledge, and limited experience in the provision of nutrition care, as well as systemic barriers like continuity of care, the poor onward referral system, and the lack of access to practicing dietitians in Ireland as significant barriers to their provision of nutrition care in routine practice. Patient and HCPs’ attitudes to nutrition care were also reported as substantial barriers to the provision of nutrition care.

“There’s a lack of access to dietetics for the families I work with and I think there’s a lack of specific support for children who have ARFID or extreme aversive feeding.”—P17 (Female, SLT, 5–10 years’ experience).

“I don’t always have the time. It’s probably the biggest one. I can be very busy with, you know, phone calls and prescriptions. It’s impossible to start and finish a task without a few interruptions”. “Yeah, time pressure is always one.”—P15 (Male, Pharmacist, 10–20 years’ experience).

Some participants iterated limited access to nutrition training and resources, while others reported easily accessible but conflicting nutrition resources.

“It’s just to get information, like where will I get the information? Where can I do the research? There are some modules at present. I think Sligo is one of them. I found one part time in Cork. But most of them are not accessible for me. I live in Tralee, so they aren’t assessable for me.”—P11 (Female, Pharmacist, 5–10 years’ experience).

“There’s so much conflicting advice on nutrition as well. There are so many, you know, diets and advice that comes up, and actually navigating through that research and dissecting facts from opinion is challenging for us and challenging for our patients.”—P9 (Female, Pharmacist, 20–30 years’ experience).

Participants also highlighted the systemic gap in access to dietitians and called for legislation and regulatory changes that would influence these systemic gaps and address the need for optimal nutrition care at all levels, especially in primary care.

“Dietitians advise separately if they get a referral, they get a pharmacist advice separately, you know that is very disjointed and also I think maybe, you know, maybe to free ourselves up to be more and of an overall holistic professional, not just and you know quick fixes with medication.”—P8 (Female, Pharmacist, 10–20 years’ experience).

Notwithstanding the barriers to the provision of nutrition care, some participants equally highlighted personal and acquired nutrition knowledge, continuing professional development (CPD) courses (some sponsored or signposted by licensing bodies such as the Irish Pharmacy Union (IPU)) and the Irish Nutrition and Dietetic Institute (INDI), working with experienced staff, access to dietitians, time, and interdisciplinary collaboration as the main facilitators to the provision of nutrition care in their routine practice.

“Definitely the articles and journals we have, some provided by IPU, the Irish Pharmacy Union, they provide us with information in articles, it’s the Pharmacy organisation. They provide webinars as well and courses and so that is what I have been very interested in and these have helped.”—P13 (Female, Pharmacist, 5–10 years’ experience).

“I suppose just my own personal knowledge, like alongside that and trying to live the healthy lifestyle myself will be the main facilitator.”—P4 (Female, Physiotherapist, 5–10 years’ experience).

Importance of the multidisciplinary and patient-centred approach in the provision of nutrition care

All participants acknowledged the importance of a multidisciplinary, holistic, patient-centred approach, which ideally necessitates the presence of a dietitian on the clinical team in addition to evidence-based resources, which would encourage the provision of nutrition care as part of routine practice in primary healthcare. Some participants reported that the optimisation of patient care prompts their quest for knowledge on nutrition and self-directed learning, which fosters interdisciplinary relations and upskilling in essential competencies, thereby contributing to the provision of holistic patient-centred care. However, this may not be the case in all primary care practices. Likewise, patients’ prompts and queries on nutrition information initiate the conversation to proffer dietary counselling.

“So getting to express your questions to much more experienced dietitians is really helpful because you know what to do if you face a similar scenario.”—P3 (Female, Dietitian, 1–5 years’ experience).

Training needs and preferences to improve competency in nutrition care

Two sub-themes were identified under this theme: (1) training needs, and (2) training delivery/preferences.

Some participants reported specific training needs on nutrition supplementation and total parenteral nutrition may be beneficial, while others reported that disease-specific training may be more beneficial due to intersections during clinical practice in areas like aversive feeding, fertility, conception, infant feeding, geriatrics, vulnerable groups, and chronic diseases.

“In my current role I think geriatric care would be a benefit, but that is the demographic that I am working with, so some type of geriatric nutrition. Not necessarily based on taking a tablet, but on getting the best nutrition from food.”—P18 (Female, Pharmacist, 10–20 years’ experience).

“I think it would make me a lot more comfortable in terms of dealing with vulnerable groups, I think just kind of up-to-date bulletins on what best practice is or what is seen to be at the moment and sort of the research around it.”—P10 (Male, Pharmacist, 5–10 years’ experience).

Participants acknowledged that nutrition-specific training alone may not be sufficient to ensure that advice is communicated adequately to patients, citing the importance of clinicians’ competencies, like communication skills, active listening skills, counselling skills, and coaching skills, being essential to ensure optimal transfer of nutrition knowledge from clinician to patient.

“Active listening skills evoke awareness facilitating the clients growth and also you know, really working with that person to support them to make one change at a time.”—P9 (Female, Pharmacist, 20–30 years’ experience).

“I think soft skills are important, how to speak to a patient, how to explain things with a simple language, adjusting medical jargon to the needs of the patient, that’s an additional skill for knowledge transfer during a conversation.”—P6 (Female, Pharmacist, 10–20 years’ experience).

All participants emphasised the inadequacy of education on nutrition in their clinical training at all levels, including undergraduate and postgraduate training. They emphasised the importance of nutrition education to support dietary counselling in practice; however, some disparity was evident in relation to the timing and mode of delivery of this ad-hoc training. Some participants argued that nutrition education ought to be integrated into undergraduate training to optimise clinicians’ foundational knowledge and understand the key principles in relation to nutrition in clinical practice. One dietitian expressed that resources and nutrition information developed by fellow dietitians may be resourceful for other multidisciplinary primary care HCPs. Some participants expressed that online courses, CPDs, diplomas, modules, and trainings may proffer the most benefit (as they are practical and self-paced) due to the demands of clinical practice. Regardless of the mode of delivery and timing, participants affirmed that nutrition education and training were essential in clinical practice. They highlighted the importance of a multidisciplinary (MDT) “buddy” approach to training and education on nutrition care, alluding to the imperative role of dietitians. A few participants cited the importance of taking responsibility with regard to self-directed learning, which is a crucial part of clinical practice. Consistently, all participants advocated for the integration of nutrition education throughout their clinical careers.

“I suppose, they could do talks or like training courses and things, you know, on site for us, maybe in the nursing home that might be relevant as in other healthcare settings, you know, like hospitals.”—P20 (Female, HCA, 5–10 years’ experience).

“We need a training based on your own experience, because one of the practitioners in my clinic, has more than 20 or 30 years’ experience, so they can provide better nutrition consultation when compared to a fresh graduate like me.”—P19 (Female, Pharmacist, 1–5 years’ experience).

“Probably they could provide nutrition knowledge or an optional subject on nutrition, within the degree (Pharmacy).”—P11 (Female, Pharmacist, 10–20 years’ experience).

Extensive primary care practice experience was associated with increased reported competency in the provision of nutrition care, as HCPs with greater than 10 years’ experience were more likely to have received additional training in nutrition. This was associated with the frequency of patients’ prompts, self-identified deficits in their own knowledge, or related to mandatory training at work. Likewise, HCPs with greater than 10 years’ experience were more likely to have obtained an additional qualification in nutrition care or dietary counselling.

“I do feel competent now in providing nutrition care because I do continuing professional development and I have diplomas and in coaching and dietary counselling and which is which gives me the continuing professional development resources and both of those to stay current with latest emerging research.”—P9 (Female, Pharmacist, 20–30 years’ experience).

“Yes, I attended courses on nutrition, and this was mainly because I had interest in this. So this was my choice. This was not anything that would be mandatory for me to do”. They were usually just like evening seminars.” “I would have a number of like shorts, evening seminars or I would read articles.”—P6 (Female, Pharmacist, 10–20 years’ experience).

Addressing barriers to the provision of nutrition counselling in primary care

Participants provided specific solutions to mitigate the barriers to the provision of nutrition care: (1) local solutions and (2) national solutions.

Participants proffered solutions that may be implemented locally to support HCPs in the provision of nutrition care, such as patient leaflets, raising awareness of local dietetic services, education for the general population, improving the quality of nutrition education provided to clinicians, and multidisciplinary support.

“If pharmacies were made aware of the local dietitian’s service or a number to contact them, it would be great.”—P12 (Male, Pharmacist, 20–30 years’ experience).

“Give people reliable sources or make it easier for them to understand, you know, rather than make it too complicated. I don’t know how much people can comprehend when you talk about nutrition, so make it easy.”—P16 (Male, Pharmacist, 1–5 years’ experience).

“Better clinical training for primary care health professionals to be able to provide general advice while waiting for access to a dietitian.”—P17 (female, SLT, 5–10 years’ experience).

Participants reported that legislative and governmental regulations are essential to optimising the integration of nutrition care into standard clinical practice, with the goal of improving patient outcomes. Participants recommended patient-centred empowerment programs by the Health Service Executive to improve national compliance with dietary recommendations.

“There’s very little support and regulation legislation for parental nutrition, it’s very ad hoc in various hospital settings, there’s no clinical guidance for pharmacists when they’re dispensing it”. “I think just the cross functionality of the healthcare system is poor at the moment.”—P5 (Female, Pharmacist, 20–30 years’ experience).

“Nutrition is a combination of nutrition and exercise and should be promoted a lot more by the HSE. I’d like to see what would happen if we had illustrations of nutritional science processes as short videos or gif-based adverts on social media platforms; or the same as part of health education in schools to see if it could make a difference to people’s behaviour, especially for visual learners.”—P15 (Male, Pharmacist, 10–20 years’ experience).

## 4. Discussion

This study examined the perceived role, barriers, facilitators, and training needs essential to the provision of nutrition care by primary HCPs in the Republic of Ireland. The 20 primary HCPs in this study affirmed that a multidisciplinary approach in patient-centred care is the underpinning, multiagency belief system for optimal nutrition care, which is consistent with existing research [33]. Participants consistently recognised the importance of nutrition care in primary care, reporting their perceived role and essential competencies, and acknowledging the barriers to the provision of nutrition care in terms of knowledge and skills. The identified themes from this study highlighted the need for a paradigm shift towards multidisciplinary training on nutrition care and integrated models of patient care. This innovative system will prioritise access to dietitians at the primary care level and provide an optimal patient referral system that supports the escalation of identified nutrition care needs. These needs may transcend the primary HCPs’ remit; however, they will suffice as gatekeepers and points of entry into the healthcare system.

Pharmacists who participated in this study referred to themselves as “gatekeepers” and the first point of contact for patients, especially in hard-to-reach areas, with difficulty in securing GP appointments. This idea is consistent with existing studies, as community pharmacists are four times more likely to engage with patients than their GPs in the primary care setting [34,35]. They are equally easily accessible, established stakeholders in health promotion and disease management through lifestyle modification, which encompasses signposting patients to receive adequate nutrition care [36,37]. In some remote parts of Northern Ireland, pharmacists may be the only ones accessible HCP [21], yet their nutrition literacy and education may be lacking [38].

Pharmacists in this study reported regularly interfacing with patients on nutrition care when prescribing or dispensing oral nutritional supplements (ONSs), which may potentially be optimised as an opportunity to initiate dietary counselling [39,40]. Nutrition interventions comprising dietary counselling alongside ONS prescriptions have been shown to be a major keystone in managing malnutrition [39,40,41,42]. Nevertheless, the inappropriate prescription of ONS due to gaps in knowledge, nutrition education, and limited competencies persists, resulting in over-prescription or under-prescription [43,44,45].

The most prominent barriers to the provision of nutrition care identified in this study were time constraints, a lack of training, and insufficient resources. These findings are consistent with previous research, highlighting the challenges of limited time during consultations, insufficient formal education in nutrition, and the scarcity of resources available to HCPs in primary care settings [46,47]. The prevalence of these practice barriers underscores the systemic nature of the challenges faced by HCPs, indicating that these issues are deeply ingrained in the healthcare infrastructure globally.

Lack of time is directly linked to a lack of resources, such as staffing, which indirectly impacts the quality of care provided by healthcare professionals across Ireland [26,47]. HCPs identified short staffing as a contributing factor to the inability to engage in meaningful conversations with patients, as they simply do not have the time. Therefore, it is seen to be extremely difficult to engage in a meaningful conversation relating to anything other than the condition they are present with [48].

The variability in levels of training and education in nutrition care and the corresponding disparity in nutrition competence are consistent with existing research [49,50,51]. The positive correlation between post-qualification training in nutrition care and self-reported competency in nutrition care, especially counselling skills [52,53], communications skills, active listening skills, and motivation was largely consistent with existing research [54,55,56]. Studies have also shown that the lack of training and education for HCPs on nutrition has manifested as a lack of confidence in dietary counselling. This is justifiably related to the patient’s perceived role of the HCPs in providing dietary counselling and/or the HCPs’ need for nutrition knowledge validation and risk aversion associated with nutrition misinformation [57,58,59]. Primary care physicians reported that increased support from legislative bodies and governmental and regulatory authorities in relation to mandatory training, policies, and health promotion initiatives centred on nutrition care may improve public health indices [56,60,61,62].

Participants acknowledged the need for post-qualification education on nutrition care. However, they reported that incorporating nutrition education at the undergraduate level may be more effective. This is consistent with previous studies, as nutrition education at undergraduate level [41,63,64,65], and post-qualification in form of online modules, courses, CPD, and nutrition materials (articles, information, and leaflets) would afford evidence-based standardised guidance to primary HCPs to provide nutrition care alongside efficient resources on nutrition to patients and service users [22,66,67,68]. Advancements have been made to integrate nutrition education in medical training with notable projects like the “Need for Nutrition Education Project” (NNedPro), which postulates and evaluates interventions in nutrition education in the UK and The American Heart Association’s Guidelines for Dietary Counselling designed for physicians in the United States [69,70,71,72].

Experiential training on behaviour change strategies and coaching skills, as well as nutrition-specific training, was recurrently emphasised as an essential clinician competency to support nutrition care as part of routine practice. This is consistent with the existing literature, as optimal communication skills are essential to explore patients’ individual needs [73,74] and sustain collaborative relationships between the multidisciplinary team and the patients [75,76]. The association between optimal communication skills and positive patient outcomes is consistent globally [67,68,69,70,71,72,73,74,75,76,77,78,79]. Competencies like active listening skills [76,80], rapport-building [81], and empathy [82] are crucial to ensure an overall positive patient experience and good quality of care [83].

A lack of resources and support was identified as a critical barrier, with participants calling for more comprehensive tools, access to dietitians, and reliable, evidence-based materials. A previous study emphasised that equipping healthcare professionals with the necessary tools for effective nutrition care will result in better referral pathways and access to dietitians [46]. Lack of access to a dietitian in primary care was mentioned as a major barrier in providing nutrition care due to inadequate remuneration. The lack of access to dietitians and the long waiting lists were equally identified as barriers. Some participants noted that complex cases may require help from different multidisciplinary practitioners, leaving dietary counselling at the bottom of the priority list. One study identified the lack of actual pathways to allied health professionals as a significant barrier to medical doctors in the UK and Australia [26]. Another study found that Irish pharmacists see the shortage of community dietitians and inadequate remuneration as significant obstacles, underscoring the need for more community dietitians [41].

Several facilitators were identified that can support HCPs in overcoming these challenges. Collaboration within multidisciplinary teams (MDTs) was identified as a critical facilitator, with the majority of participants highlighting the benefits of working closely with dietitians and other healthcare professionals. The literature supports this finding, which consistently shows that collaborative care models improve patient outcomes by allowing for more comprehensive and coordinated care [41]. Access to dietitians, in particular, was seen as a positive influence on one’s ability to provide dietary care within their role. Working with dietitians in the acute setting was mentioned as a significant facilitator, identifying the importance of interdisciplinary learning. The importance of interprofessional nutrition practices and the positive outcomes that can lead to working collaboratively with other healthcare professionals to achieve a common goal cannot be overemphasised [84].

Personal barriers, such as patient engagement and role limitations, pose challenges to delivering effective dietary counselling within current healthcare roles and regulations. Existing studies reported that practice nurses faced difficulties due to patients’ lack of interest and motivation towards nutrition, which hindered meaningful discussions about dietary changes [46,85].

A paradigm shift to a multidisciplinary care model in delivering nutrition care has significantly improved in specialist areas like end-of-life care [86] and chronic conditions [87], but this care model is yet to be adopted in primary care to promote the delivery of nutrition education and dietary counselling [88,89,90]. There is evidence to show that dietary interventions at community pharmacies have promoted this interdisciplinary care model in prioritising nutrition care, thereby adding value and fostering health promotion [91,92]. This MDT approach may support health promotion strategies and successful interventions in maternal and child health, weight management, hyperlipidaemia, improve glycaemic control, and optimise community-based management of hypertension [21,22,30]. Concomitant with the existing literature, participants echoed the challenge of access and referral pathways to dietitians [25], albeit their perceived role was signposting and referring patients, they acknowledged the gaps in multidisciplinary care.

The importance of a holistic and systemic approach to dietary counselling was also emphasised, with participants noting the value of considering the broader context of a patient’s life, including family dynamics, personal preferences, and habits. This approach aligns with the principles of patient-centred care, which advocate for a more individualised and context-specific approach to healthcare [93]. One study found that tailoring advice specific to an individual enabled healthcare professionals to provide more effective dietary counselling [84].

Access to reliable resources and a personal interest in nutrition were identified as key facilitators in providing effective dietary counselling. Participants with access to trustworthy, evidence-based information and who engaged in self-directed learning felt better equipped for dietary counselling. Participants acknowledged that the majority of the nutrition materials available in primary care are provided by pharmaceutical and nutrition companies, which may constitute bias [41,88]. This calls for more research to standardise nutrition education and training materials for primary HCPs in practice. Research has emphasised that personal interest motivates exploration and learning, enhancing environmental engagement [94]. Those with a strong interest in nutrition were likelier to engage in meaningful conversations, pursue continued learning, and feel more confident discussing nutrition.

Patients have reported that doctors were more trustworthy and motivating if they practised healthy lifestyles and demonstrated evidence-based experience in motivational counselling techniques to improve nutrition and health behaviours. They reported that nutrition care may be more impactful when practitioners are equipped to provide individualised care [95]. Likewise, patients living with obesity found GP and practice nurses’ advice to be crucial motivators of their weight loss journey, highlighting the beneficial role of GP resources and the applicability of GP-led behaviour change interventions [96,97,98]. Similar studies have found positive correlations between the inclusion of the 5As (ask/assess, advise, agree, assist, and arrange) framework [11] for the management of obesity in the curricula and CPD programs of medical students, residents, practising physicians, and other HCPs. The studies presented positive correlations between nutrition training, ranging from 15 min to 25 h, and improvements in patients’ nutritional status [11], physical activity levels, weight management, weight loss behaviours [99], and behaviour change [100]. These curricula were based on theories such as Social Cognitive Theory [100], Motivational Interviewing [101], The Transtheoretical Model of Behaviour Change [102], and The RE-AIM Framework [103]. However, recent studies that illustrate a direct association between primary HCP training in nutrition and improved health literacy and chronic disease management are lacking.

To the best of our knowledge, this is the first study to examine the perceived roles and training needs of primary healthcare professionals in a single cohort within the Republic of Ireland, contributing significantly to the body of knowledge. Semi-structured interviews are a reliable way to obtain data on self-perceived roles and competencies. This study was evidently limited by the generalisability of the findings, as 70% of the participants were pharmacists, which is not a sufficient representation of the primary care professional distribution in Ireland. The overwhelming response and participation of pharmacists may be due to access granted by the PSI (The Pharmacy Society of Ireland) to the repository of registered pharmacists in the Republic of Ireland. Following their data protection guidelines, the researchers received access to the pharmacists’ contact details and utilised these in the recruitment process. Three participants were dietitians, which may have impacted the interpretation of data on the roles of primary HCPs in the provision of nutrition care. Likewise, there was poor engagement from other regulatory and licensing organisations in a bid to reach their primary care professionals.

## 5. Conclusions

The findings from this research suggest that primary HCPs are key stakeholders in providing optimal nutrition care as crucial points of contact and entry into the healthcare system, thereby mitigating the gap created by a lack of access to dietitians in the Republic of Ireland. A plausible range of approaches includes optimal nutrition education systems, an integrated multidisciplinary model of care, consistency in nutrition care delivery, and standards of dietary counselling. Facilitators like personal clinical experiences and access to dietitians are often mitigated by a lack of nutrition knowledge and time constraints.

However, an amalgamation of local and national solutions may directly address the barriers to the provision of nutrition care in primary practice. Further research on the feasibility of integrating nutrition education in the clinical curricula of primary HCPs in the Republic of Ireland, the development of post-qualification training modules, and quality control measures to ensure evidence-based, safe nutrition care is provided to all patients and service users is required. Likewise, further research, including other members of the primary care team, is critical.

## Figures and Tables

**Table 1:** Summary of participants’ demographic data collated from the online questionnaire.

Characteristics	% (n)
Number of participants	n = 20
**Gender**	
Male	25 (5)
Female	75 (15)
**Highest Level of Education**	
Undergraduate	20 (4)
Postgraduate Diploma	10 (2)
Masters	70 (14)
**Primary Healthcare Profession**	
Pharmacist	70 (14)
Healthcare Assistant	5 (1)
Dietitian	15 (3)
Speech and Language Therapist	5 (1)
Physiotherapist	5 (1)
**Years of primary healthcare practice experience (years)**	
1–5	25 (5)
5–10	35 (7)
10–20	20 (4)
20–30	15 (3)
30+	5 (1)
**Working part-time or full-time**	
Part-time	40 (8)
Full-time	60 (12)

## Data Availability

The data presented in this study are available on request from the corresponding author.

## Supplementary Materials

The following supporting information can be downloaded at https://www.mdpi.com/article/10.3390/ijerph22050771/s1, Interview questions and Coreq checklist.
