# Supplementary material for: Enhancing Nutrition Care in Primary Healthcare: Exploring Practices, Barriers, and Multidisciplinary Solutions in Ireland
Source: Int J Environ Res Public Health. 2025 May 13;22(5):771. doi: 10.3390/ijerph22050771 (PMC12111206; doi:10.3390/ijerph22050771)
Supplement: Supplementary file 1 [file ijerph-22-00771-s001.zip › ijerph-3553177-supplementary.pdf]

SUPPLEMENTARY MATERIALS

Interview Questions

<table>
<tr><td>Interview Questions</td><td>Enquiry Probe</td></tr>
<tr><td colspan="2">Logic for Q's 1 - 2: To explore the scope and perceived role of primary healthcare professionals in the provision of dietary counselling in practice</td></tr>
<tr><td>1). As a primary healthcare practitioner, do you provide dietary counselling? I.e., individualised nutritional care and advice for your patients
Probe:
• If yes, what for?
• What is the scope of dietary counselling you provide? (Personalized counselling or general dietary counselling?)
• If yes, how many times do you provide this advice? (Daily, weekly, monthly).
• If no, what barriers hindered you from providing this service?</td><td>To see if it is something which a person in their role carries out as part of their job.</td></tr>
<tr><td>2). What is your perceived role in offering nutrition care/ dietary counselling?
Probe:</td><td>Do they agree or disagree that nutrition care is part of their role in providing patient care or do they believe it is someone else's role.</td></tr>
</table>

• Do you feel obligated to provide dietary counselling as part of your routine practice?<br>• Do you feel competent to provide dietary information?	
Logic for Q's 3-5: To explore the level of training received/ acquired by primary healthcare practitioners on nutrition in their clinical training,	
3). Do you think the competencies (knowledge, skills, and attitudes) relating to the provision of nutrition care vary across different primary healthcare providers? - **Probe:**<br>• - If yes, in what way?<br>• Do you believe there was sufficient focus on dietary counselling and nutrition in your clinical training?	Do they believe that some HCP are better equip than others in providing nutrition care to patients
4.) Do you believe there was sufficient focus on dietary counselling and nutrition in your clinical training? Probe:<br>• If yes, give details.	To gain an insight into their training in college in relation to nutrition care.
5) Have you received any additional training in nutrition while in your current role and if so what kind of additional training did you receive and was it beneficial? E.g., Continuous professional development	To gain an insight into how many HCP have furthered their knowledge in relation to nutrition education or is it something that could be brought in as a CPD course.

**Probe:**<br><br>• If yes, give details (CPD, Certificate course, Postgraduate diploma, Masters, PhD)<br>• If yes, when did you receive this training? (Immediately after qualification, during practice) If no training, what training do they feel would be beneficial for providing nutrition care in their current role?	
Logic for Q's 6-7: To explore the perceived facilitators to the provision of nutrition care, including gaps, competencies and how best these can be addressed/ acquired/ improved.	
6) In your experience, can you identify the facilitators to the provision of nutrition care?<br>**Probe:**<br><br>• What factors have supported you in your provision of dietary counselling?<br>• What other factors do you think may support you in your provision of dietary counselling?	To identify the main facilitators within that group of HCP.
7)In your scope of practice, what competencies do you think are important for you to deliver nutrition care?<br>**Probe:**	What that group of HCP perceive as important competencies for providing nutrition care.

<table>
<tr><td>Based on these competencies, do you think any gap exists in your clinical training? Competent in nutrition information? Competent in discussing nutrition with patients? Competent in your knowledge of nutrition in relation to a persons condition?
How best can these gaps be addressed? (CPD, extra modules in training curriculum)</td><td></td></tr>
<tr><td colspan="2">Logic for Q’s 8-9: To explore the perceived Barriers identified by the HCP in the provision of providing accurate nutrition Care to patients within the Primary Care Setting</td></tr>
<tr><td>8). What do you believe are the Main Barriers in providing nutrition care to patients under your care?</td><td>To identify the main barriers</td></tr>
<tr><td>9). How do you think these Barriers could be addressed?

Probe
If it is relating to lack of education surrounding nutrition counselling, ask how they believe an HCP knowledge could be enhanced in the field of nutrition care.</td><td>To identify how barriers could be addressed</td></tr>
</table>

10). Do you have any additional thoughts or opinions on this subject that you would like to share?	

COREQ (COnsolidated criteria for REporting Qualitative research) Checklist [31]

Topic	Item No.	Guide Questions/ Description	Reported on Page No.
**Domain 1: Research team and reflexivity**			
Personal characteristics			
Interviewer/facilitator	1	Which author/s conducted the interview or focus group?	22
Credentials	2	What were the researcher's credentials? E.g. PhD, MD	2
Occupation	3	What was their occupation at the time of the study?	2

Gender	4	Was the researcher male or female?	N/A
Experience and training	5	What experience or training did the researcher have?	N/A
Relationship with participants			
Relationship established	6	Was a relationship established prior to study commencement?	21
Participant knowledge of the interviewer	7	What did the participants know about the researcher? e.g. personal goals, reasons for doing the research	21
Interviewer characteristics	8	What characteristics were reported about the inter viewer/facilitator? e.g. Bias, assumptions, reasons and interests in the research topic	21
**Domain 2: Study design**			
Theoretical framework			
Methodological orientation and Theory	9	What methodological orientation was stated to underpin the study? e.g. grounded theory, discourse analysis, ethnography, phenomenology, content analysis	23
Participant selection			
Sampling	10	How were participants selected?	

		e.g. purposive, convenience, consecutive, snowball	21
Method of approach	11	How were participants approached? e.g. face-to-face, telephone, mail, email	21
Sample size	12	How many participants were in the study?	24
Non-participation	13	How many people refused to participate or dropped out? Reasons?	24
Setting			
Setting of data collection	14	Where was the data collected? e.g. home, clinic, workplace	24
Presence of non-participants	15	Was anyone else present besides the participants and researchers?	22
Description of sample	16	What are the important characteristics of the sample? e.g. demographic data, date	25
Data Collection			
Interview guide	17	Were questions, prompts, guides provided by the authors? Was it pilot tested?	22
Repeat interviews	18	Were repeat inter views carried out? If yes, how many?	N/A
Audio/visual recording	19	Did the research use audio or visual recording to collect the data?	22
Field notes	20	Were field notes made during	22

		and/or after the inter view or focus group?	
Duration	21	What was the duration of the inter views or focus group?	24
Data saturation	22	Was data saturation discussed?	22
Transcripts returned	23	Were transcripts returned to participants for comment and/or	23
**Domain 3: analysis and findings**			
Data analysis			
Number of data coders	24	How many data coders coded the data?	23
Description of the coding tree	25	Did authors provide a description of the coding tree?	26
Derivation of themes	26	Were themes identified in advance or derived from the data?	26
Software	27	What software, if applicable, was used to manage the data?	23
Participant checking	28	Did participants provide feedback on the findings?	N/A
Reporting			
Quotations presented	29	Were participant quotations presented to illustrate the themes/findings? Was each quotation identified? e.g. participant number	15-25
Data and findings consistent	30	Was there consistency between the data presented and the findings?	27-37
Clarity of major	31	Were major themes clearly	26

themes		presented in the findings?	
Clarity of minor themes	32	Is there a description of diverse cases or discussion of minor themes?	27
